# A dataset examining the impact of direct electronic medical record interfacing on the accuracy of point-of-care urinalysis results

**DOI:** 10.1016/j.dib.2023.109012

**Published:** 2023-03-01

**Authors:** Kai J. Rogers, Matthew D. Krasowski

**Affiliations:** Department of Pathology, University of Iowa Hospitals and Clinics, Iowa City, IA 52242, USA

**Keywords:** Clinical laboratories, Clinical laboratory information systems, Clinical laboratory services, Medical errors, Point-of-care testing

## Abstract

Point-of-care testing is widely used in a variety of clinical settings. While this testing provides immediate and actionable clinical information, it is prone to error in both the interpretation and reporting of results. Point-of-care urinalysis presents unique opportunities for errors, ranging from variation in visual interpretation to input of results. The data included here represent the results from 63,279 urinalyses from 36,780 unique patients performed over a span of three years at an academic medical center and its associated clinics. The data include the patient age/legal sex, methodology (instrument and test strip used), and the available test results (color, clarity, glucose, bilirubin, ketones, specific gravity, blood, pH, protein, urobilinogen, nitrite, and leukocyte esterase). Additionally, we include the method of interface between the testing instrumentation and our electronic medical record (EMR). These fell into one of three broad categories: “Interfaced” (results directly transmitted from the urinalysis instrument to the EMR via specialized data interface), “Manual” (results input by selecting from a drop-down menu in the laboratory information system), and “Enter/Edit” (results typed freely into a text field in the EMR). Analysis of this data was primarily a direct comparison of detectable errors (typos, uninterpretable results, and results outside the reportable range) as a function of the method of entry into the EMR. Secondary analysis comparing the impact of restricting drop-down menu options for urine color and clarity was also performed. These data are of use to others as they are diverse in terms of the test performed and the method of interface. Others may wish to analyze these data when making decisions as to how to perform and report these tests and when estimating risks of error with various methods of data entry.

**Specifications Table**

**Table:** 

Subject	Medicine and Dentistry
Specific subject area	Pathology and Medical Technology
Type of data	Tables Figures Supplemental data
How the data were acquired	Retrospective chart and data review from laboratory analyses performed at an academic medical center and its affiliated clinics were obtained via tools within the electronic medical record (EMR; Epic, Inc.) covering the time period from January 1, 2019 through July 9, 2021. The data were focused on point-of-care urinalysis testing and included some combination of the following urinalysis components: color, clarity, glucose, bilirubin, ketones, specific gravity, blood, pH, protein, urobilinogen, nitrite, and leukocyte esterase. Of note, we included all samples which had point-of-care urinalysis performed within the retrospective timeframe and did not exclude any data from downstream analysis except those with an issue such as insufficient specimen or incorrect collection that resulted in test cancellation. The project had approval as a retrospective study with waiver of informed consent from the University of Iowa Institutional Review Board (protocol # 202107265).
Data format	Raw and Analyzed
Description of data collection	The data included point-of-care urinalysis performed from January 1, 2019 through July 9, 2021. There was a total of 63,279 urinalysis panels from 36,780 unique patients (28,320 female, 8460 male), some of whom had data in more than one category of result entry. The data included 30,300 urinalysis orders from interfaced results, 29,197 from manually resulted results, and 3782 from Enter/Edit. The inclusion criteria include point-of-care urinalysis results entered into the EMR by interface, manual drop-down, or Enter/Edit during the retrospective timeframe. Tests were excluded only if a result was not available due to an issue such as insufficient specimen or incorrect collection that resulted in test cancellation.
Data source location	University of Iowa Hospitals and Clinics, Iowa City, Iowa, United States of America
Data accessibility	The raw dataset of this article is provided as Microsoft Excel file (.xlsx) and can be found in Mendeley repository data. https://data.mendeley.com/datasets/7wy7b8hjz7/3[1] 3 tables and 3 figures are included in the paper.

## Value of the Data

• This data set highlights the common challenges and pitfalls associated with reporting point-of-care urinalysis test results to an electronic medical record.
• The data is beneficial to clinicians, medical professionals, and laboratory personnel that either collect or interpret urinalysis tests or report their results.
• These data could be used by clinicians and clinical laboratory leadership to determine the best approach to testing in their practice.
• This is a large and diverse data set that may be of use to researchers interested in the performance of urinalysis testing or assessing the potential impact of data interfacing on error rates.

## 1 Objective

Point-of-care testing is used in a variety of medical settings, and results ideally should be high-quality and accessible in the EMR [2], [3], [4]. Approaches used to deposit point-of-care data in the EMR include electronic interface of point-of-care instrument results to the EMR, manual entry of results into the EMR or laboratory information system using drop-down menus or similar methods to select from choices, and completely manual (free text) data entry [5], [6], [7]. For urinalysis, variability can occur from inter-operator differences in interpretation of color and clarity of the urine along with differences in reading dipstick color changes for other urinalysis parameters such as glucose or ketones [8], [9], [10], [11], spurring interest in novel technologies to reduce bias of colorimetric analysis [12]. Possible errors from manual entry include misspellings, flipping or transposing results, and numerical entry errors [6,7,9,11]. Some manual entry errors are unlikely to result in patient harm, such as misspelling of a word that is still recognizable. However, numeric errors or flipping results can more easily result in patient harm depending on clinical context. This data set was generated to investigate the frequency and nature of data entry errors as a function of the testing platform and interface method used. One limitation of our study is that the analysis did not capture manually entered results that may have been different from the actual point-of-care result but were plausible and in the correct format for the analyte being reported. This more subtle type of error has been investigated in a study of glucose meter results where both interfaced and manually entered results were inadvertently simultaneously reported during a transition to result interfacing [6]. In the present data set, variability of results recorded in the EMR can occur from a combination of factors including result entry method (e.g., manual vs. interface), operator interpretation, and instrument performance.

## 2 Data Description

**Fig. 1:**
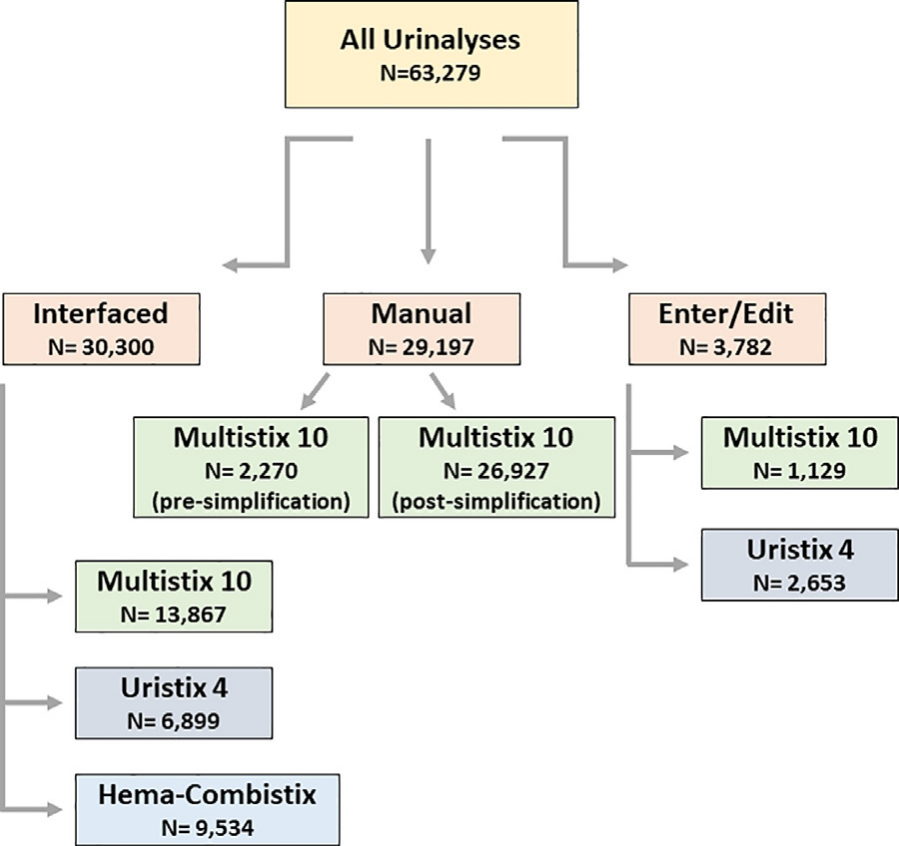
Data outline.

**Fig. 2:**
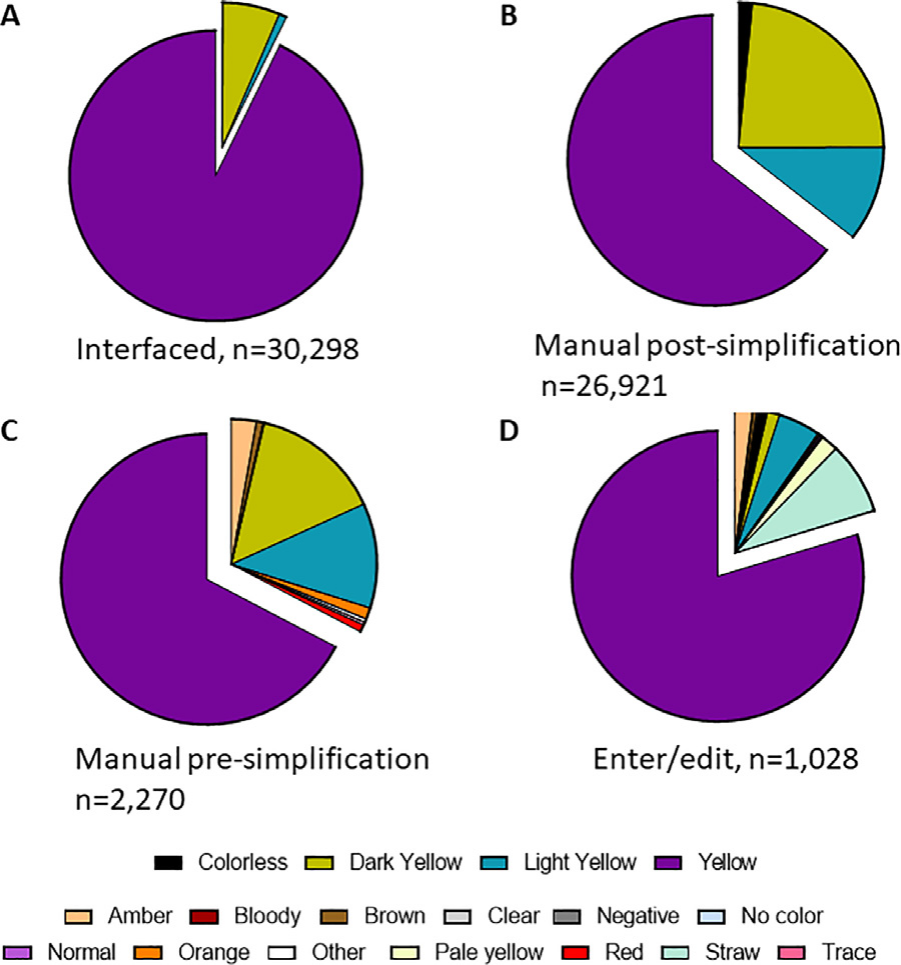
Range of reported values for urine color.

**Fig. 3:**
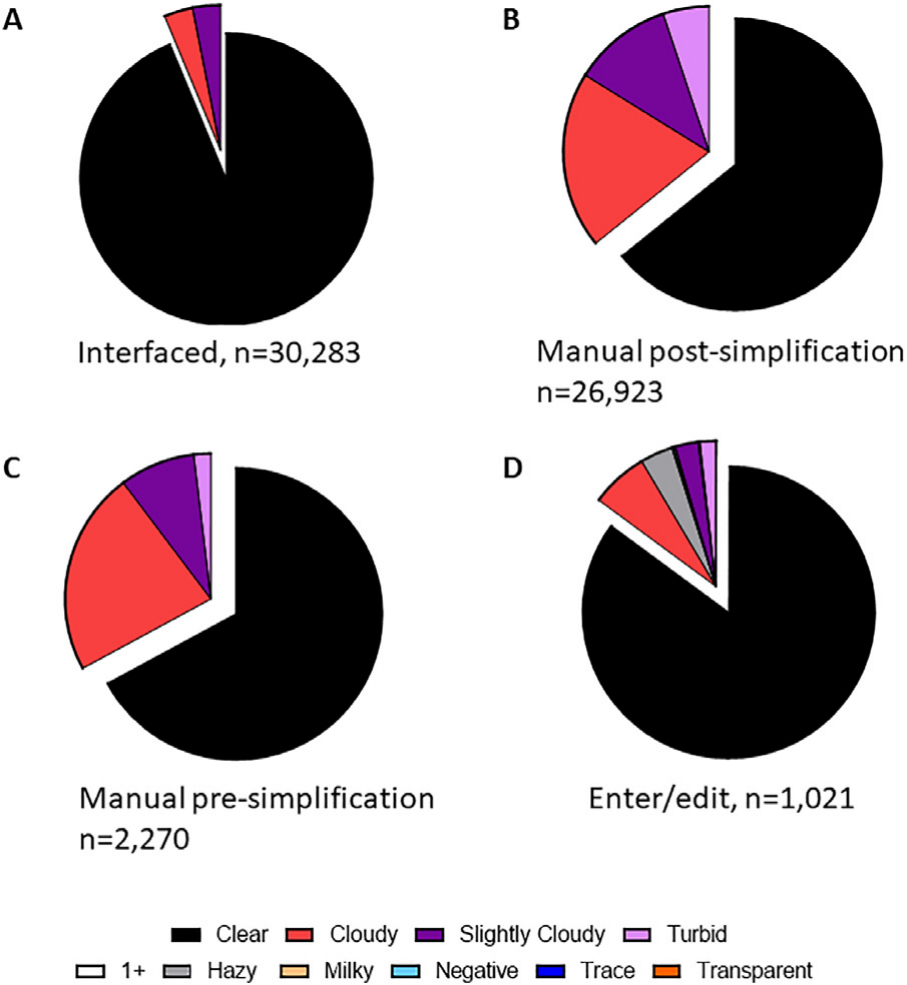
Range of reported values for urine clarity.

Retrospective data between January 1, 2019 and June 9, 2021 in this study was acquired from the EMR by reporting tools. Fig. 1 and Table 1 show the breakdown of data used in the study. Fig. 2 shows differences in results for urine color depending on method of result entry. Fig. 3 shows differences in results for urine clarity. Table 2 shows number of unique entries seen in data for urinalysis result components depending on method of result entry. Table 3 summarizes types of errors seen in data depending on method of result entry.

**Table 1:** Datasets included in the study.

	Data Transmission/Entry Method^a^			
	Interfaced	Manual – Pre-Simplification	Manual – Post- Simplification	Enter/Edit
Number of urinalysis tests	30,300	2270	26,927	3782
Number of unique patients (female / male)	16,342 (12,762/3580)	1,407 (1,076/331)	13,616 (11,749/1867)	1,692 (1306/386)
Age range in years	0.0, >89	0.0, >89	0.0, >89	0.1, >89
Mean age ± SD	36.8 ± 19.3	39.3 ± 19.1	39.7 ± 19.7	36.7 ± 26.1

a Description of these categories is provided in the manuscript.

**Table 2:** Number of unique entries seen in data for urinalysis result components.

	Data Transmission/Entry Method^a^			
Urinalysis Component	Interfaced	Manual – Pre-Simplification	Manual – Post- Simplication	Enter/Edit
Color	5	10	5	22
Clarity	5	4	7	14
Glucose	6	5	7	24
Bilirubin	5	4	4	15
Ketones	7	6	12	24
Specific gravity	7	9	8	49
Blood	6	5	5	31
pH	9	11	22	17
Protein	6	5	5	43
Urobilinogen	6	5	6	23
Nitrite	3	2	2	13
Leukocyte esterase	6	5	5	22

a Description of these categories is provided in the manuscript. Note that following the switch to “Manual Post-Simplification”, there was a brief period of time where the result selected by drop-down result could be further manually edited. There was thus a limited number of typos (e.g., “Cloudy0” instead of “Cloudy” for Clarity) seen for this reason. This functionality was removed.

**Table 3:** Summary of errors investigated in the study.

	Data Transmission/Entry Method			
Urinalysis Component (number, % of total)	Interfaced	Manual – Pre-Simplification	Manual – Post-Simplification	Enter/Edit
Typographical errors	2 (< 0.001%)^1^	0 (0%)	6 (0.002%)^a^	19 (0.1%)
Outside reportable range	0 (0%)	0 (0%)	0 (0%)	13 (0.06%)
Uninterpretable	0 (0%)	0 (0%)	15 (0.005%)^b^	316 (1.4%)

a Four of these errors (2 Interfaced, 2 Manual) stemmed from ability to manually change spelling of entry after it had been transmitted by interface or selected manually in dropdown menu. This capability has since been removed, while retaining the ability to error correct a result to another dropdown choice, if appropriate.

b All of these involved data entry errors in pH, which is typed in manually (not a drop-down as opposed to many of the other urinalysis components) for the Manual method.

Fig. 1 shows a graphical representation of the data included in the study. Of note, the range of options provided in the “Manual” data entry method changed during the retrospective timeframe. Options for urine color and clarity were restricted to a smaller number of choices in October 2019 and thus these data were analyzed separately (“Manual Pre-Simplification” prior to October 2019 and then “Manual Post-Simplification” for manual data entry results thereafter).

For Fig. 2, specimens were organized by the method of interface with the EMR. Categories include “Interfaced” data for which the operator selects color of sample using the instrument software (with color options shown on screen) which is then transferred by interface to the EMR, “manual entry” data which are interpreted by the collecting provider and then entered into the EMR through a series of drop-down menus that restrict the range of accepted values, and finally “Enter/Edit” data which are interpreted at the point of collection and then typed into a “free-text” box that does not restrict the range of entries. The spectrum of resulted values is displayed as pie charts with the number of specimens in each category indicated below. Of note, the range of options provided in the “manual entry” method for urine color were restricted in October 2019 and thus these data were analyzed separately (“Manual Pre-Simplification” prior to October 2019 and then “Manual Post-Simplification” for manual data entry results thereafter). For simplicity, redundant categories with alternate spelling/abbreviations were combined (e.g., light yellow and lt. yellow).

For Fig. 3, specimens were organized by the method of interface with the EMR. Categories include “Interfaced” data for which the operator selects clarity of sample using the instrument software which is then transferred by interface to the EMR with no further human intervention, “Manual Entry” data which are interpreted by the collecting provider and then entered into the EMR through a series of drop-down menus that restrict the range of accepted values, and finally “Enter/Edit” data which are interpreted at the point of collection and then typed into a free-text box that does not restrict the range of entries. The spectrum of resulted values is displayed as pie charts with the number of specimens in each category indicated below. Of note, the range of options for urine clarity provided in the “manual entry” method were restricted in October 2019 and thus these data were analyzed separately (“Manual Pre-Simplification” prior to October 2019 and then “Manual Post-Simplification” for manual data entry results thereafter). For simplicity, redundant categories were combined (e.g., “slightly cloudy” and “sl. Cloudy”). As noted in Table 2, there were a limited number of errors seen in the “Manual Post-Simplification” data due to the ability to edit a result selected by drop-down (e.g., altering to “Cloudy0” instead of “Cloudy”). This functionality was subsequently removed shortly after the transition.

Table 1 contains a summary of specimens included in our data set. Of note, the range of options provided in the “manual entry” method for urine color and clarity were restricted starting in October 2019 and thus these data were analyzed separately.

Table 2 indicates the number of unique entries for each urinalysis parameter results that were identified in the indicated data set. This includes minor misspellings that would likely still be interpretable as well as other errors such as uninterpretable entries or quantitative values outside the reportable range that would be more likely to cause misinterpretation. Of note, the range of options for urine color and clarity provided in the “manual entry” method were restricted in October 2019, and thus these data were analyzed separately.

Table 3 summarizes the prevalence of the investigated error types in our data set. More detailed description of the variety of result entries is provided in the Supplementary File 1, Tab A.

**Supplementary File 1: Dataset with raw data**

Raw data file subdivided into five tabs (available at https://data.mendeley.com/datasets/7wy7b8hjz7/3)[1]

Data included are organized according to the method of interface with our EMR.

• Tab A: Key for classes of errors used in Tabs C through F and examples of these error types.
• Tab B: Interfaced choices for the urinalysis results.
• Tab C: “Interfaced” – Urine color and clarity are interpreted by the operator, with the choices selected within the urinalysis instrument software and then transmitted by interface to the EMR. The remaining urinalysis results are read by the instrument and directly interfaced with the EMR electronically. Errors are typed according to key in Tab A.
• Tab D: “Manual Pre-simplification” – These results are interpreted by the staff performing the test and entered into the EMR by selecting options from a series of drop-down menus. These tests were performed between January 2019 and September 2019. In October 2019, we restricted the number of options available in the provided drop-down menus to simplify data reporting and improve consistency. Errors are typed according to key in Tab A.
• Tab E: “Manual – Post-Simplification” – These data are similar to “Manual Pre-simplification” but occurred after the simplification of drop-down menu options. Errors are typed according to key in Tab A.
• Tab F: “Enter-Edit” – These results are interpreted by the staff performing the test and then manually entered into the EMR using free text without character restrictions or drop-down menus. Errors are typed according to key in Tab A.

For Tabs C through E, the following data are given when available:

• Urinalysis panel – the testing performed, either “Multistix 10”, “Uristix 4”, or “individual component.” See Methods for further details.
• Age – rounded to the nearest year (90 years or older reported as > 89).
• Legal sex – male, female, or unknown.
• Urinalysis results – color, clarity, glucose, bilirubin, ketones, specific gravity, blood, pH, protein, urobilinogen, nitrite, leukocyte esterase.
• Error type – uses numeric code (0, no error; 1, typo; 2, outside reportable range; 3, uninterpretable) for each urinalysis result. Error type for each urinalysis result are in separate columns.

## 3 Experimental Design, Materials and Methods

### 3.1 Data source

All retrospective data in this study was acquired via Epic Reporting Workbench, a built-in tool which facilitates extraction of data from the EMR [5]. Data were extracted between January 1, 2019 and June 9, 2021 using a component search for results associated with point-of-care urinalysis that were manually input into the EMR using drop-down choices or interfaced from the instrument. Point-of-care urinalysis results that used Enter/Edit were retrieved by search for test results that included free text. Data was obtained from patients receiving care at the University of Iowa Hospitals and Clinics (Iowa City, Iowa, USA) or affiliated practices/clinics at various locations throughout the state. No laboratory testing other than that ordered for clinical management of patients was included in the study.

### 3.2 Point-of-care testing

Urinalyses were performed using the Clinitek Status Plus (Siemens Healthineers, Cary, NC) [13] with one of the following test strips:

• **Multistix 10 SG** (Siemens Healthineers) – glucose, bilirubin, ketones, specific gravity, blood, pH, protein, urobilinogen, nitrite, and leukocyte esterase.
• **Uristix 4** (Siemens Healthineers) – glucose, protein, nitrite, and leukocyte esterase.

### 3.3 Classification of errors

Errors were identified by manual review of the data set by both authors independently and then compared to reach a consensus. This was done by first filtering out acceptable values for each test result (using the default filter function in Microsoft Excel) and then classifying the type of errors identified into the categories below.

“Typos” were defined as errors that did not change the interpretation of the data (spelling errors, single character substitutions, “++” instead of 2+, etc.).

Errors “outside reportable range” were defined as values that fell outside of the reportable range defined in the EMR that were not due to simple typographical error. As an example, the reportable range for specific gravity was 1.005 to 1.030 for the methods used in the study. Thus, a value of 1.035 entered in the EMR would be outside the defined reportable range for the specific gravity component and should have been reported as “> 1.030”.

“Uninterpretable” errors were defined as those which were not interpretable either because they were so severely misspelled or because they conveyed no clinical meaning (i.e., “normal” for a quantitative variable, “trace” for clarity, “increased” for urobilinogen, or a numeric value for a qualitative variable, etc.). Categories were mutually exclusive and thus no single error is represented more than once.

## Funding

This research did not receive any specific grant from funding agencies in the public, commercial, or not-for-profit sectors.

## Ethics statement

Institutional ethical clearance was from the University of Iowa Institutional Review Board (protocol # 202107265) as a retrospective study with waiver of informed consent. The research was carried out in accordance with the Declaration of Helsinki.

## CRediT Author Statement

**Kai Rogers:** Data curation, Data Analysis, Investigation, Writing – original draft preparation; **Matthew Krasowski:** Conceptualization, Data curation, Data Analysis, Writing – review & editing.

## Declaration of Competing Interest

The authors declare that they have no known competing financial interests or personal relationships that could have appeared to influence the work reported in this paper.

## Data Availability

Interfacing of Point-of-Care Urinalysis Results and Errors (Original data) (Mendeley Data).
